# Intraoperative capsule protection can reduce the potential risk of adjacent segment degeneration acceleration biomechanically: an in silico study

**DOI:** 10.1186/s13018-024-04550-0

**Published:** 2024-02-16

**Authors:** Fei Huang, Gang Huang, Junpengli Jia, Shihao Lu, Jingchi Li

**Affiliations:** 1 Department of Orthopedics, Meishan Hospital of Traditional Chinese Medicine, Meishan, China; 2 Department of Orthopedics, The Affiliated Traditional Chinese Medicine Hospital, Southwest Medical University, No. 182, Chunhui Road, Longmatan District, Luzhou, 646000 Sichuan Province People’s Republic of China; 3 Department of Orthopedics, Changzheng Hospital Affiliated to the Naval Medical University, Xiangyin Road, Shanghai, 200433 People’s Republic of China; 4 Luzhou Key Laboratory of Orthopedic Disorders, Southwest Medical University, No. 182, Chunhui Road, Luzhou, 646000 Sichuan Province People’s Republic of China

**Keywords:** Zygapophyseal joint, Capsule, Motion segment, Biomechanical deterioration, Iatrogenic capsule injury, Posterior lumbar interbody fusion

## Abstract

**Background:**

The capsule of the zygapophyseal joint plays an important role in motion segmental stability maintenance. Iatrogenic capsule injury is a common phenomenon in posterior approach lumbar interbody fusion operations, but whether this procedure will cause a higher risk of adjacent segment degeneration acceleration biomechanically has yet to be identified.

**Methods:**

Posterior lumbar interbody fusion (PLIF) with different grades of iatrogenic capsule injury was simulated in our calibrated and validated numerical model. By adjusting the cross-sectional area of the capsule, different grades of capsule injury were simulated. The stress distribution on the cranial motion segment was computed under different loading conditions to judge the potential risk of adjacent segment degeneration acceleration.

**Results:**

Compared to the PLIF model with an intact capsule, a stepwise increase in the stress value on the cranial motion segment can be observed with a step decrease in capsule cross-sectional areas. Moreover, compared to the difference between models with intact and slightly injured capsules, the difference in stress values was more evident between models with slight and severe iatrogenic capsule injury.

**Conclusion:**

Intraoperative capsule protection can reduce the potential risk of adjacent segment degeneration acceleration biomechanically, and iatrogenic capsule damage on the cranial motion segment should be reduced to optimize patients’ long-term prognosis.

## Introduction

Lumbar degenerative disease (LDD) is a common disease in elderly patients [1, 2]. With the increasing aging tendency in our country, the population base of the disease is step expanding [3, 4]. Degenerative changes in intervertebral disks (IVD) and zygapophyseal joints (ZJ) are the main pathological changes in LDD patients, and biomechanical deterioration initially triggers degeneration of these structures [5, 6]. Posterior approach lumbar interbody fusion (PLIF) is an effective method for the treatment of LDD [7, 8]. Adjacent segment disease (ASD) is a common postoperative complication. Related clinical symptom recurrence and revision surgery are the main triggers for poor long-term prognosis and social–economic burden [9, 10]. As a special kind of LDD, biomechanical deterioration caused by improper intraoperative procedures is also the initial trigger for ASD [9, 11]. In contrast, the optimization of surgical strategies may effectively reduce the risk of ASD by alleviating biomechanical deterioration [12, 13].

The lumbar spine is a complex structure consisting of various active and passive motion structures. The capsule of ZJ is an important structure for restricting the motion ranges in a special motion segment [14, 15]. Therefore, damage to the capsule may trigger segmental instability and resulting degeneration acceleration [16, 17]. To full exposure the pedicle screw insertion point, some surgeons completely destroy the dorsal side of the capsule [18, 19]. Based on above foundations, we hypothesize that iatrogenic capsule injury may trigger a higher risk of ASD, but this topic has yet to be identified in published studies. To verify this hypothesis, the biomechanical significance of capsule protection in the PLIF operation was validated by numerical mechanical simulations. To ensure the credibility of computed results, the model used in surgical simulations was calibrated and validated by comparing computed stress and motility characteristics values and mechanical tested values in published studies. To our knowledge, this was the first study to identify this topic.

## Material and methods

### Construction of the intact model

**Fig. 1:**
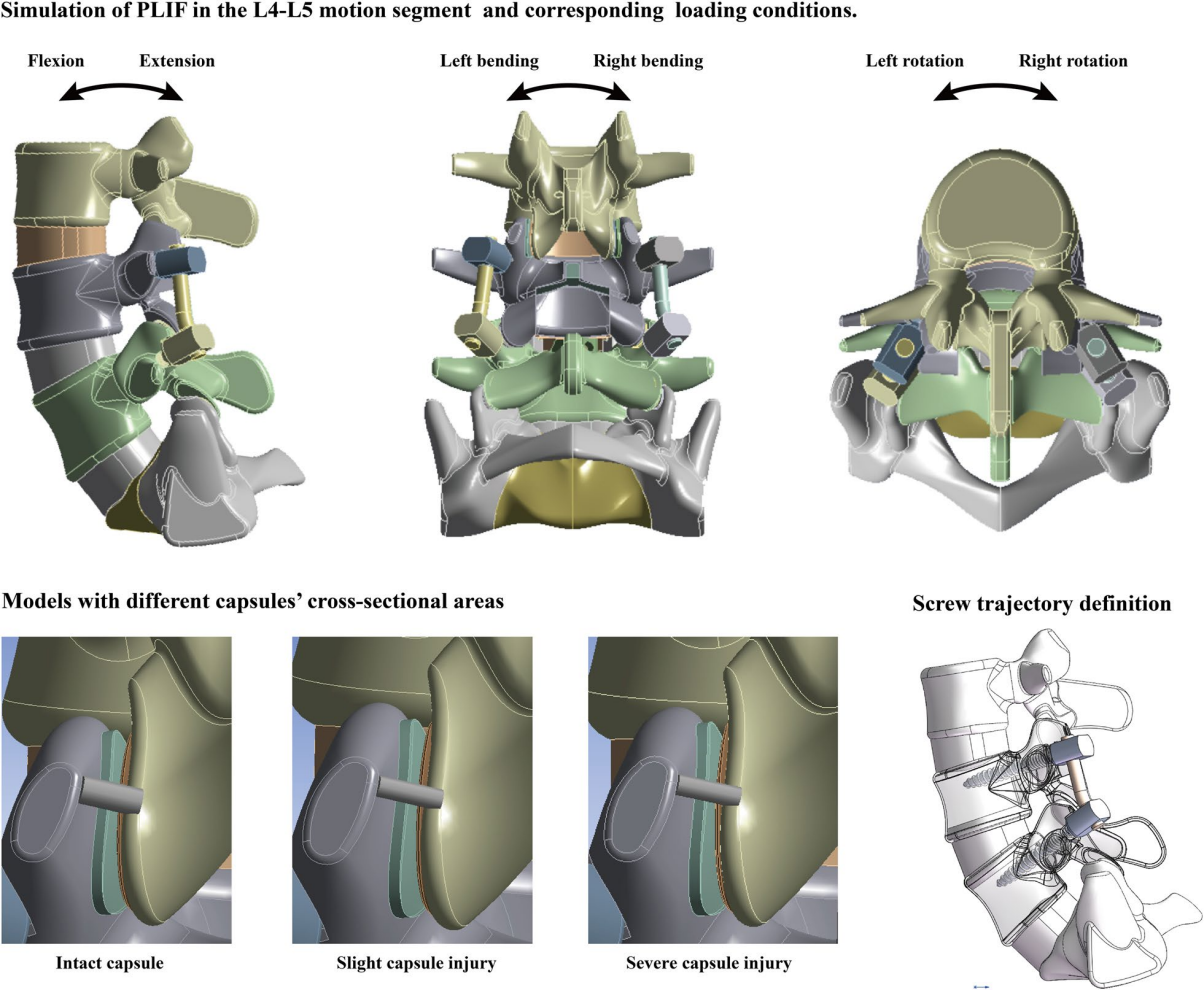
Schematic for PLIF simulations, loading conditions, different grades of capsule damage, and screw trajectories

Simulations of PLIF with different grades of iatrogenic capsule injury have been performed in our previously constructed, calibrated, and validated numerical model. The detailed model construction, calibration, and validation strategy have been well described in these studies [20, 21]. Overall, the main purpose of these procedures was to improve the computational reliability, which is reflected in various aspects [22, 23]. First, the irregular surface of the reconstructed model was completely replaced by regular surfaces in the current spinal model. By using this method, the incidence of computational error is significantly reduced [20, 21]. When constructing bony structure models, the thickness of cortical shell was defined as 0.8 mm, and the only exception was bony endplates (BEPs). Concave angles and depth of BEPs have been defined according to imaging and anatomical studies [24–26]. Moreover, when it comes to the construction of non-bony component numerical models, IVD was consisted by annulus, nucleus, and cartilage endplates (CEP). The outline of the BEP covers entire cranial and caudal surfaces of IVD, while that of the CEP covers the nucleus and the inner half part of the annulus (Fig. 1) [27–29].

### Material property definition and model calibration

Mechanical material properties of different components were defined separately [30–32]. The cortical and cancellous bones were defined using the anisotropic law, while the remaining components were considered isotropic materials. Given that the stiffness of the ligaments was highly adjustable, a significant individual difference in this parameter existed, which was selected as the model calibration parameter. By adjusting the stiffness of ligaments under different loading conditions, the computed range of motion (ROM) is prone to the average ROM value from mechanical tests [33, 34]. Moreover, given that ligamentum structures suffer large deformations, the construction of solid element ligamentum models may trigger a high incidence of computational error. Therefore, line bodies were selected to construct ligament models, including all ligaments and the capsule of the articular process.

### Multi-indicators model validation

**Fig. 2:**
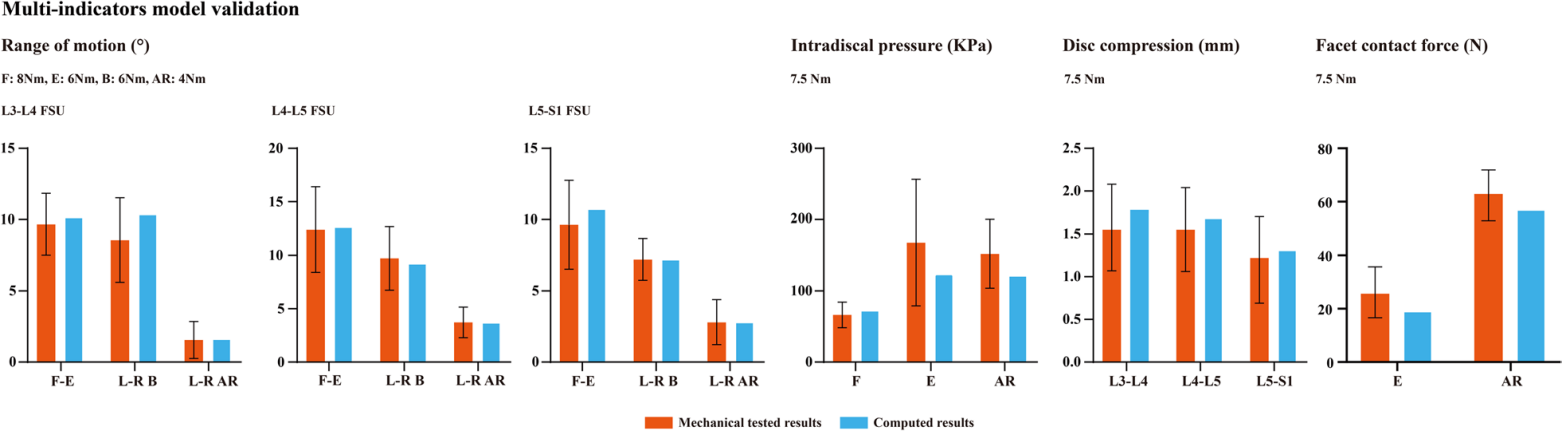
Multi-indicators model validation (consisted to our published studies)

Given that the motility characteristics were a main perspective of the biomechanical environment, this calibration strategy could optimize the computational stability. In addition, multi-indicator model validation was also performed in this model. Consistent with the model calibration process, computational results from the calibrated model were also compared to the mechanical test results. The ROM, disk compression (DC) value, facet contact force (FCF), and intradiscal pressure (IDP) were compared between the computed and tested models [35–37]. When the difference between the computed and tested values was less than one standard deviation of the mechanical test, we believe that the current model was well validated. Finally, studies show that the size of elements will affect the computational result. To eliminate this confounding effect, mesh convergence tests were also performed in the validated model. The IDP value was selected as the reference of this test. When the difference in IDP was smaller than 3% under different mesh sizes, we judged that the mesh convergence test was accomplished [22, 23]. In summary, in our published studies, we perform above-mentioned procedures to optimize the computational reliability of current numerical models (Fig. 2).

### PLIF simulations with different grades of capsule iatrogenic injury

The simulation of the PLIF operation was performed by referring to the same type of studies and our clinical experience. Specifically, consistent with our published studies, the L4–L5 motion segment was selected in the PLIF simulation for the highest incidence of lumbar degenerative disease in this segment [6, 38, 39]. When simulating nerve structure decompression, the spinous process, the lower two-thirds of the laminae, the medial third of the inferior articular process of the L4 vertebral body, and the supraspinous and interspinous ligaments were excised [40, 41]. The posterior part of the annulus and all nuclei in the L4–L5 motion segment were excised to simulate discectomy, and the cartilage endplate of both the cranial and caudal sides of L4–L5 was also excised to simulate the endplate preparation process [8, 41].

To simulate the pedicle screw fixed interbody fusion operation, bilateral pedicle screws were inserted into the L4 and L5 vertebral bodies. When constructing pedicle screw models, the screw tulip and nut were simplified to a simple structure [42, 43]. By using this method, the contact between these two structures can be simplified to achieve simplification of the model and reduce the computational burden. Consistent with our published studies, the screw trajectory was parallel to the superior bony endplate in the corresponding vertebral body on the sagittal plane and parallel to the axis of the pedicle on the transverse plane [42, 43]. In addition, the elastic modulus of bony structures around the screw trajectory was adjusted. The range of adjusted bony structures consisted of the volume of screw. To eliminate the confounding effect caused by thread preservation, all threads were completely inserted into bony structures. When simulating cage insertion, a 26-mm cage filled with bone tissue was inserted into the interbody space from the right side of the interbody space. The material properties of bone tissue were defined according to our published studies [22, 30, 44]. Finally, as mentioned above, line bodies were selected to construct capsule models. Therefore, the cross-sectional area of the capsule was reduced to simulate different grades of intraoperative iatrogenic capsule injury. One-fourth and one-half cross-sectional area reduction of the L3–L4 capsule (the motion segment cranial to the surgical segment) were performed to construct PLIF models with slight and severe capsule injury (Fig. 1).

### Boundary and loading conditions

The boundary and loading conditions of ASD value computation consisted of the computation of ROM in the model calibration process. By selecting this model computation strategy, the computational reliability can be effectively ensured. Specifically, the inferior surfaces of the PLIF models were completely fixed under all degrees of freedom, and different directional moments, including 8-Nm flexion, 6-Nm extension, left and right bending, and 4-Nm left and right directional axial rotation, were applied on the superior surface of L3 (Fig. 1) [22, 23]. The craniofacial coefficient between bone–screw interfaces and cage–bone interfaces was 0.2, and that between grafted bone and bony endplates was 0.46 [22, 23].

## Results

**Fig. 3:**
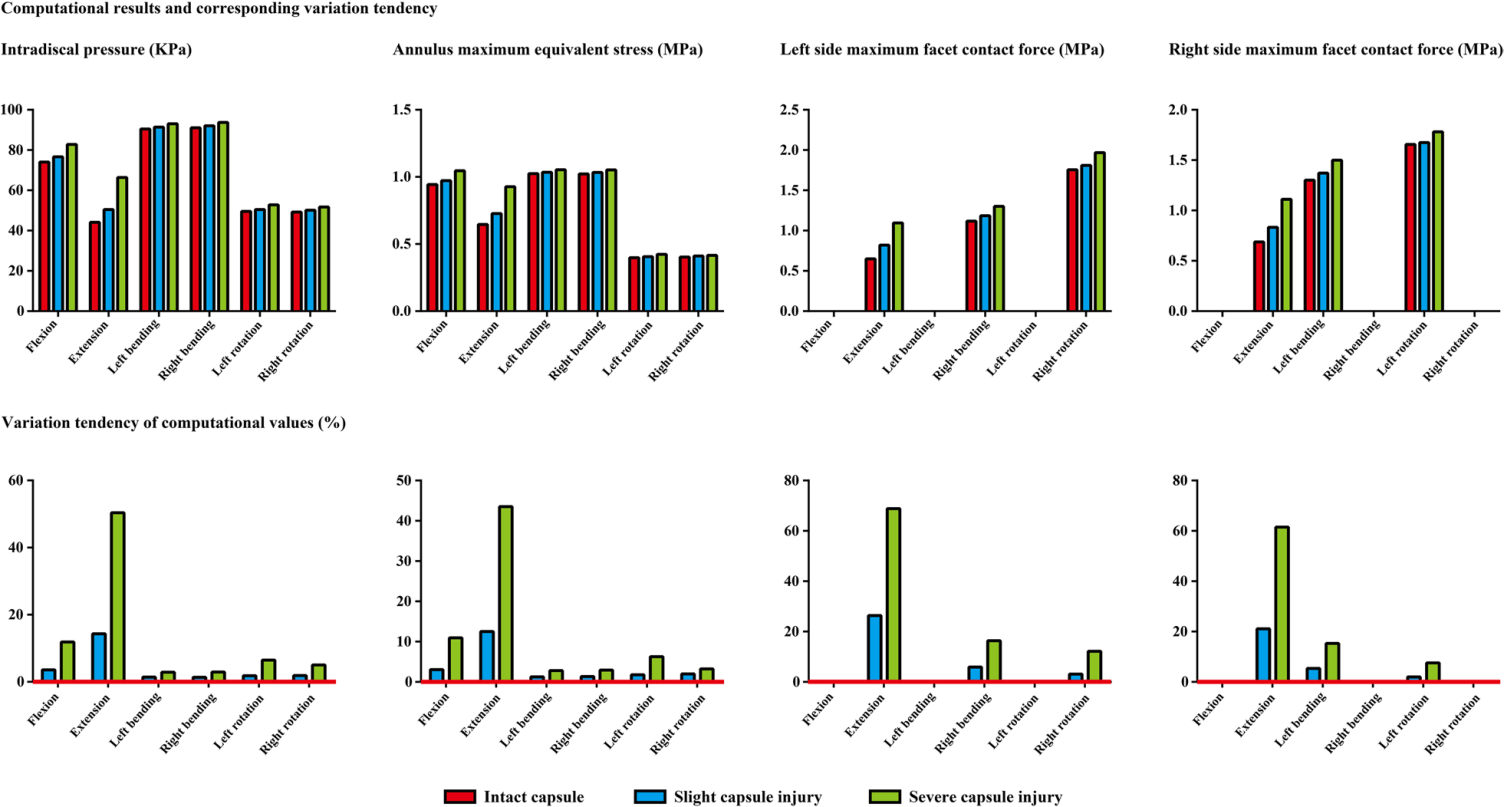
Computational results and variation tendency caused by capsule damage

**Fig. 4:**
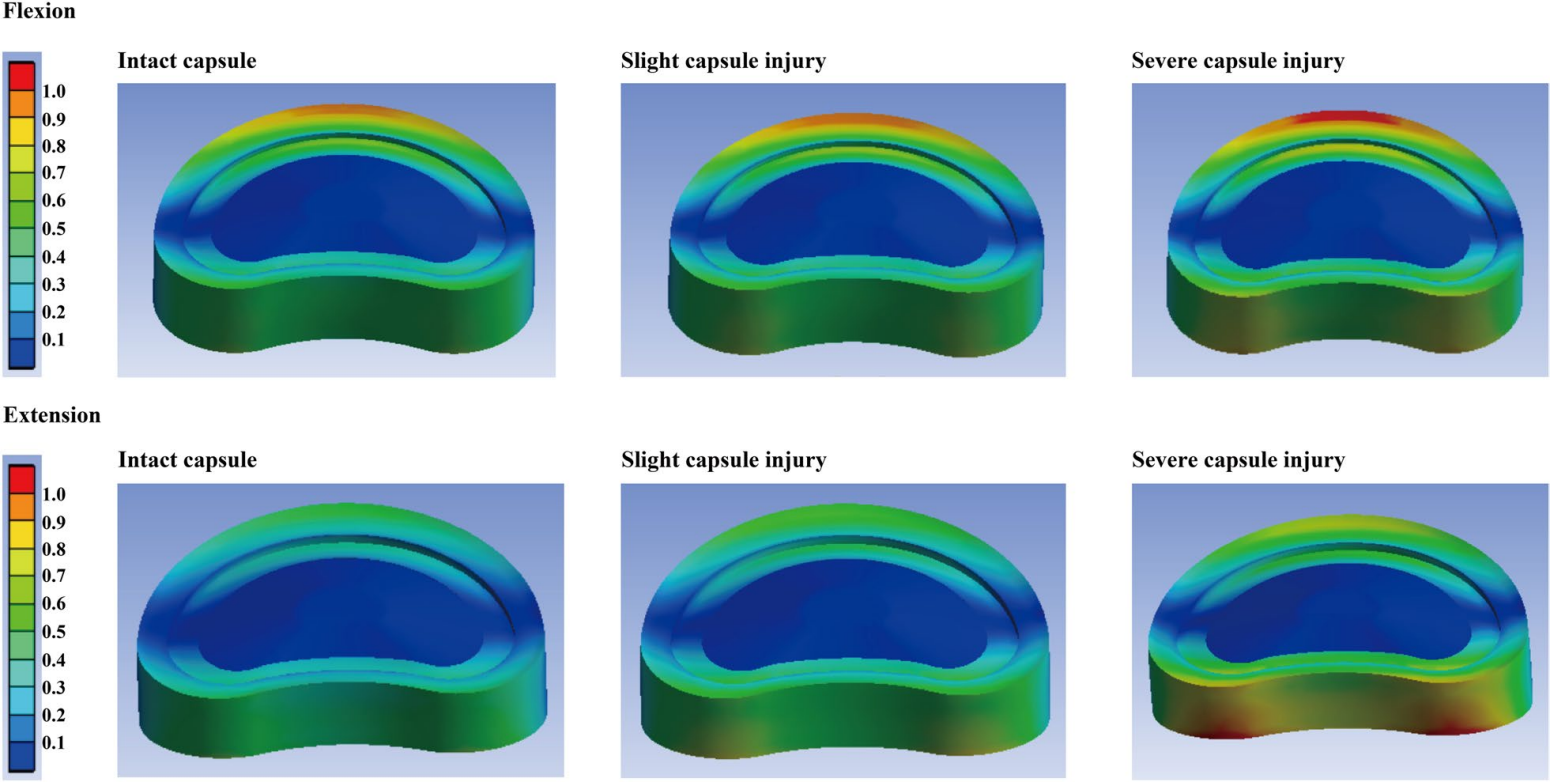
Nephograms of annulus distribution under flexion and extension loading conditions

To evaluate the potential risk of cranial motion segmental ASD, IDP, FCF, and the maximum stress on the annulus were computed and recorded in this study. It is worth noting that under bending and rotation loading conditions, only facet contact on the contralateral side can be recorded. In other words, only the right-side FCF can be recorded and vice versa. A similar variation tendency of the computational result can be observed under almost all loading conditions. Specifically, a higher stress value can be observed with a reduction in the capsule cross-sectional area. In this process, a slight biomechanical deterioration can be recorded in the model with slight capsule injury, and obvious stress concentration can also be recorded in the model with one-half capsule cross-sectional area reduction. More significantly, compared to the difference between models with slight intact capsule and capsule injury, the difference between models with slight and severe capsule injury is more obvious. The most obvious biomechanical change can be observed under the extension loading condition. Compared to the model with an intact capsule, the IDP value in the model with severe capsule injury increased by more than 50%, the maximum annulus equivalent stress increased by 40%, and the value of FCF even increased by 60% (Figs. 3 and 4, Tables 1, 2, and 3).

**Table 1:** Computed intradiscal pressure in different models (KPa)

	Intact capsule	Slight capsule injury	Severe capsule injury
Flexion	74	76.66	82.77
Extension	44.15	50.47	66.39
Left bending	90.45	91.37	93.05
Right bending	91.05	92.01	93.71
Left rotation	49.55	50.45	52.76
Right rotation	49.2	50.12	51.67

**Table 2:** Computed annulus maximum stress in different models (MPa)

	Intact capsule	Slight capsule injury	Severe capsule injury
Flexion	0.943	0.972	1.046
Extension	0.646	0.727	0.927
Left bending	1.024	1.034	1.053
Right bending	1.022	1.033	1.052
Left rotation	0.398	0.405	0.423
Right rotation	0.403	0.411	0.416

**Table 3:** Computed left FCF in different models (MPa)

	Intact capsule	Slight capsule injury	Severe capsule injury
*Flexion*			
Extension	0.648	0.819	1.094
*Left bending*			
Right bending	1.118	1.184	1.301
*Left rotation*			
Right rotation	1.755	1.809	1.968

## Discussion

Posterior approach lumbar interbody fusion is a common surgical strategy to treat LDD patients [19, 45]. The incidence of facet joint injury and the damage of ZJ capsule were nearly one-half in the pedicle insertion process [46, 47]. In which, intraoperative iatrogenic capsule injury is common to expose the bone structure around entry point of the pedicle screw. Considering that the capsule is an important structure of segmental stability maintenance, and segmental instability is a main reason for segmental degeneration acceleration, this procedure may trigger a higher risk of ASD in the cranial motion segment biomechanically after the lumbar interbody fusion operation. To verify this hypothesis, PLIF models with different grades of capsule injury were constructed based on a well-validated model constructed in our published studies, and stress values related to ASD were computed and recorded in this study.

The results show that capsule injury will lead to the deterioration of the stress value of the corresponding motion segment and trigger a higher risk of ASD biomechanically. More importantly, only a slight biomechanical deterioration can be observed in the model with slight capsule injury (Figs. 3 and 4, Tables 1, 2, and 3). Based on the current computational results, although it may be impractical to completely avoid intraoperative capsule injury (capsules may obscure the entry point of the pedicle, especially in patients with articular process hypertrophy), reducing the extent of capsule injury is still recommended to alleviate cranial segmental biomechanical deterioration and corresponding risk of ASD. Moreover, given that the most dramatic stress concentration can be recorded under the extension loading condition, this study also proves that the biomechanical significance of the capsule, and even of ZJ, is most significant under this loading condition. This conclusion was consistent with the same type of studies [48, 49].

The following aspects should be clarified from the methodological perspective. First, only stress values on the cranial motion segment were recorded in this study. This is because the incidence of cranial ASD was significantly higher than that of caudal ASD [9, 50]: Surgical segmental high stiffness is the main reason for ASD biomechanically. Compared to the caudal side, given that the pedicle screw was more prone to the cranial position, more obvious stress concentration can be observed on the cranial side after the PLIF operation [12, 50, 51]. More significantly, from the clinical perspective, intraoperative injury of the caudal side capsule is not necessary. Therefore, these factors should not be investigated in this study (Table 4).

**Table 4:** Computed right FCF in different models (MPa)

	Intact capsule	Slight capsule injury	Severe capsule injury
*Flexion*			
Extension	0.688	0.833	1.111
Left bending	1.301	1.371	1.5
*Right bending*			
Left rotation	1.656	1.674	1.781
Right rotation			

IDP, annulus maximum stress, and FCF were computed and recorded to represent potential risk of ASD. Stress concentration on the annulus and higher IDP values was proved to be risk factors for annulus tears [52, 53]. In the lumbar spine, annulus tear is the main phonological phenotype for IVD degeneration and LDD progression [6, 15]. Considering higher annulus stress and IDP values can be observed in models with iatrogenic capsule injury, we can deduce that this procedure may trigger higher risk of IVD degeneration and resulting ASD. In addition, FCF has also been computed to judge ASD risk. This is because the pathological process of ASD is not limited to degeneration of the intevertebral disk, acceleration degeneration of ZJ, and corresponding spinal canal stenosis, which is also an important pathological type of ASD [54, 55], especially for elderly patients (an epidemiological study reported that spinal canal stenosis, rather than lumbar disk herniation, is the main reason for lumbar surgery in elderly patients) [41, 55, 56]. In a word, by comprehensively computing IDP, annulus stress, and FCF, the current numerical models can good represent potential risk of ASD biomechanically.

Besides, pure moments, rather than moments with compressive load, were applied on current models for following reasons. Firstly, there are large individual differences in the amount of compressive load, and which was significantly influenced by weight of patients [32, 44]. In contrast, moment is a relatively constant indicator of lumbar motion [31, 39]. More significantly, calibration and validation process of the current numerical model was accomplished under pure moments [20, 21]. The computational credibility of the current study can be ensured by computing mechanical parameters under the loading condition with model calibration and validation.

Meanwhile, endplate damage was also an important reason for intervertebral disk degeneration, but stress distribution on the endplate was not recorded in his study. Acute trauma is the main reason for endplate damage and the resulting acceleration of IVD degeneration. However, the loading conditions selected in this study were to simulate those in patients’ daily lives [6, 15]. Therefore, endplate damage will not occur in this injury type. Finally, only one-half cross-sectional area reduction was selected when simulating severe capsule injury. When performing posterior approach lumbar surgery, the capsule on the ventral side cannot be injured; therefore, in the severe capsule injury model, one-half of the capsule cross-sectional area was reduced to simulate complete injury of the dorsal side capsule. In summary, the basic mechanism of ASD and the real situation of intraoperative situations were the main references for the currently selected model construction strategy.

Admittedly, the following limitations still existed in this study. First, capsule suffered large deformation under current loading conditions, solid element with large deformation values suffers high incidence of computational error [22, 31]. Therefore, line bodies, rather than solid elements, were selected for capsule model construction to ensure analysis convergence [33, 57]. Limited by this model construction strategy, the biomechanical significance of the capsule in different special regions cannot be identified in current models. We will try to perform numerical simulations by using different analysis modules, such as the dynamics computational module, to further verify the current computational results. In addition, our previous surgical records did not record the grade of capsule injury in detail and were limited by the ethical principle of clinical practice. Thus, the current biomechanical research conclusion cannot be validated by prospective clinical studies. The lack of clinical evidence is an important limitation of this study. Although these limitations existed, given that a consistent and obvious variation tendency can be observed in current models under different loading conditions, we still believe that the current research conclusion is reliable and should be revalidated in our future studies with further optimized models.

## Data Availability

All the data of the manuscript are presented in the paper.
